# The Role of Alpha 6 Integrin in Prostate Cancer Migration and Bone Pain in a Novel Xenograft Model

**DOI:** 10.1371/journal.pone.0003535

**Published:** 2008-10-28

**Authors:** Tamara E. King, Sangita C. Pawar, Lisa Majuta, Isis C. Sroka, Danyel Wynn, Manolis C. Demetriou, Raymond B. Nagle, Frank Porreca, Anne E. Cress

**Affiliations:** 1 Department of Cell Biology & Anatomy, The Arizona Cancer Center, University of Arizona, Tucson, Arizona, United States of America; 2 Department of Pharmacology, The Arizona Cancer Center, University of Arizona, Tucson, Arizona, United States of America; 3 Department of Physiology, The Arizona Cancer Center, University of Arizona, Tucson, Arizona, United States of America; 4 Department of Biological Sciences, University of Cyprus, Nicosia, Cyprus; 5 Department of Pathology, The Arizona Cancer Center, University of Arizona, Tucson, Arizona, United States of America; Dresden University of Technology, Germany

## Abstract

Of the estimated 565,650 people in the U.S. who will die of cancer in 2008, almost all will have metastasis. Breast, prostate, kidney, thyroid and lung cancers metastasize to the bone. Tumor cells reside within the bone using integrin type cell adhesion receptors and elicit incapacitating bone pain and fractures. In particular, metastatic human prostate tumors express and cleave the integrin A6, a receptor for extracellular matrix components of the bone, i.e., laminin 332 and laminin 511. More than 50% of all prostate cancer patients develop severe bone pain during their remaining lifetime. One major goal is to prevent or delay cancer induced bone pain. We used a novel xenograft mouse model to directly determine if bone pain could be prevented by blocking the known cleavage of the A6 integrin adhesion receptor. Human tumor cells expressing either the wildtype or mutated A6 integrin were placed within the living bone matrix and 21 days later, integrin expression was confirmed by RT-PCR, radiographs were collected and behavioral measurements of spontaneous and evoked pain performed. All animals independent of integrin status had indistinguishable tumor burden and developed bone loss 21 days after surgery. A comparison of animals containing the wild type or mutated integrin revealed that tumor cells expressing the mutated integrin resulted in a dramatic decrease in bone loss, unicortical or bicortical fractures and a decrease in the ability of tumor cells to reach the epiphyseal plate of the bone. Further, tumor cells within the bone expressing the integrin mutation prevented cancer induced spontaneous flinching, tactile allodynia, and movement evoked pain. Preventing A6 integrin cleavage on the prostate tumor cell surface decreased the migration of tumor cells within the bone and the onset and degree of bone pain and fractures. These results suggest that strategies for blocking the cleavage of the adhesion receptors on the tumor cell surface can significantly prevent cancer induced bone pain and slow disease progression within the bone. Since integrin cleavage is mediated by Urokinase-type Plasminogen Activator (uPA), further work is warranted to test the efficacy of uPA inhibitors for prevention or delay of cancer induced bone pain.

## Introduction

Of the estimated 565,650 people in the U.S. who will die of cancer in 2008, almost all will have metastasis[1]. Breast, prostate, kidney, thyroid and lung cancers metastasize to the bone. The tumor cells within the bone elicit osteolytic and osteoblastic reactions and incapacitating bone pain and fractures[2], [3]. One major goal is to prevent or delay cancer induced bone pain. Invasive and metastatic human prostate tumors express integrin A6B1, a receptor for extracellular matrix components of the bone, i.e., laminin 332 and laminin 511[4]–[10].

Human prostate cancer is an indolent disease characterized by progressive adhesion changes during the transition from normal glands to prostatic intraepithelial neoplasia (PIN) to invasive cancer[5], [16]–[19]. Recent work has shown alteration in the normal human prostate tissue organization and adhesion molecules during prostate tumor progression[5], [15]. Escape from the prostate gland and invasion through the capsule is associated with poor prognosis whereas confined disease is treatable. Alterations in adhesion molecules and the downstream signaling consequences may account for the stimulated invasion of tumor cells from their site of origin. Integrins are transmembrane heterodimer cell adhesion receptors [15]. Integrin expression within the normal prostate gland reflects the diversity of the extracellular matrix components. Normal patterns of integrin expression are maintained in lesions in which normal basal cells retained and invasion has not occurred (i.e., PIN lesions) [5], [16]–[19]. However, within invasive carcinomas, the majority of the integrin subunits are not observed on the tumor cell surfaces. A notable exception to the pervasive loss of integrin expression is persistent expression of the laminin receptors, A3 (10% of cases) and A6 (69% of cases) integrins, observed in the invasive human prostate carcinoma obtained after radical prostatectomy [5], [17]. Studies indicate that the laminin receptors A6B1 and A3B1 are maintained in the majority of prostate carcinomas. During the human PIN to prostate carcinoma transition, A6B4 integrin expression is lost and A6B1 integrin predominates in invasive human prostate cancer and in metastatic lesions[4], [6]. Numerous studies have implicated the A6 integrins in cancer progression[20]. The extracellular ligands for A6B1 are laminin 332 and 511, prominent constituents of human and mouse bone marrow[7], [21].

An inspection of the A6B1 integrin expression on prostate tumor cells reveals a novel structural variant on the cell surface called A6pB1[11], [14], [22]. The A6 integrin subunit is cleaved in half at the tumor cell surface at specific amino acid residues resulting in loss of the beta barrel domain and leaving the rest of the receptor intact[12]. Prostate tumor cells expressing a mutated receptor that cannot be cleaved, resulted in an inhibition of tumor cell migration on laminin 332 under tissue culture conditions[13].

In this study, we determined if integrin cleavage would affect tumor cell migration within a clinically relevant metastasis site, such as bone. We used a novel xenograft mouse model of bone pain to directly determine the effects of human tumor cells placed within the living bone matrix on cancer induced bone pain. The bone marrow is rich in laminin 332 (Laminin 5) and laminin 511 (Laminin 10)[8], [9], the ligands for the A6B1 integrin[7]. We further tested the influence on cancer induced bone pain since previous work strongly implicated integrins in the maintenance of neuropathic pain[23].

## Results

**Figure 1:**
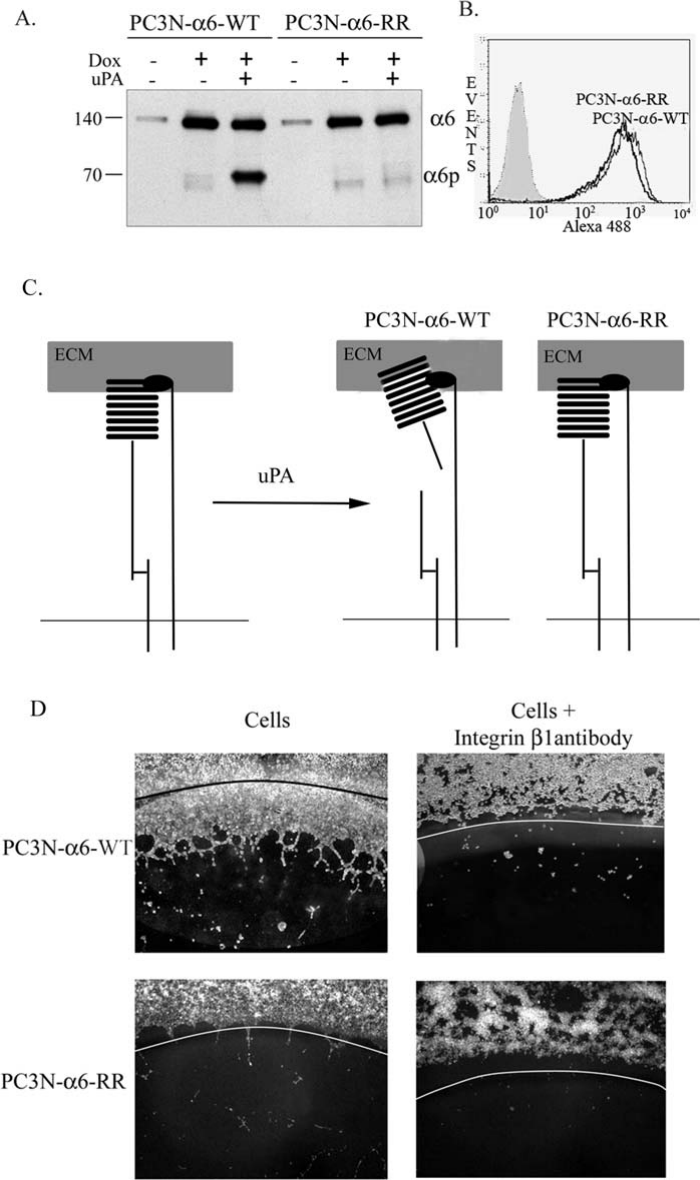
Biochemical and migration phenotype of PC3N-A6-WT and PC3N-A6- RR cells expressing the wildtype(cleavable) and RR(uncleavable) integrin A6, respectively. (A) The expression of the full length 6 integrin ( 6) and uPA dependent production of the 6p variant ( 6p) was determined by western blot analysis. Integrin status within total cell lysates from doxycycline (Dox) or urokinase (uPA) untreated (−) and treated (+) PC3N-A6-WT and PC3N-A6-RR cell lines was determined. (B) Surface expression of wild type and mutated integrin 6 in doxycycline induced PC3N-A6-WT and PC3N-A6-RR cells was determined by flow cytometry. PC3N-A6-WT and PC3NA6- RR cells were incubated with primary Rat anti-integrin A6 antibody J1B5 followed by Alexa 488 anti-rat antibody and visualized using the BD FACScan. The grey peak indicates fluorescence signal from secondary antibody only. (C) Schematic to illustrate the cleavage of the full length form of the 6 integrin to yield the 6p variant. The definition of the PC3N-A6-WT and PC3N-A6-RR cells with regard to integrin status is shown. (D) Integrin mediated migration of PC3N-A6-WT cells (top panels) and PC3NA6- RR cells (bottom panels) on matrigel. The cells were placed on matrigel in the presence of a coverslip to create a cell free zone on the matrigel surface. After cell adhesion was complete, the coverslips were removed from the matrigel surface to allow migration into the cell free zone indicated by white or black curved line. Cells migration occurred under optimal growth conditions at 37 for approximately 18 hours. Cells were either allowed to migrate in the absence (left panels) or presence (right panels) of integrin blocking antibody AIIB2. Images were collected using a Zeiss Axiovert microscope equipped with a 2.5X objective.

Accordingly, we used the PC3N-A6-WT and PC3N-A6-RR prostate cancer cells that express equivalent levels of the wild-type A6 subunit (cleavable to A6p via uPA treatment) and the non-cleavable subunit, respectively (Fig. 1a). The surface expression levels of the receptors were equivalent as determined by Flow Cytometry (FACS) analysis (Fig. 1b). The ability to cleave the receptor by Urokinase-type Plasminogen Activator (uPA) and the generation of the A6p structural variant is shown schematically (Fig. 1c). We next tested the functional properties of A6 integrin using a laminin 111 containing matrix, matrigel, modified to contain laminin 332. Matrigel is a laminin rich extracellular matrix that models physiologically relevant conditions[24]. The PC3N-A6-WT cells migrated within matrigel in a manner that was integrin dependent (**Fig. 1d**). The cells containing the uncleavable receptor, PC3N-A6- RR, were unable to migrate in matrigel, consistent with previous results using routine tissue culture conditions (**Fig. 1d**)[13].

**Figure 2:**
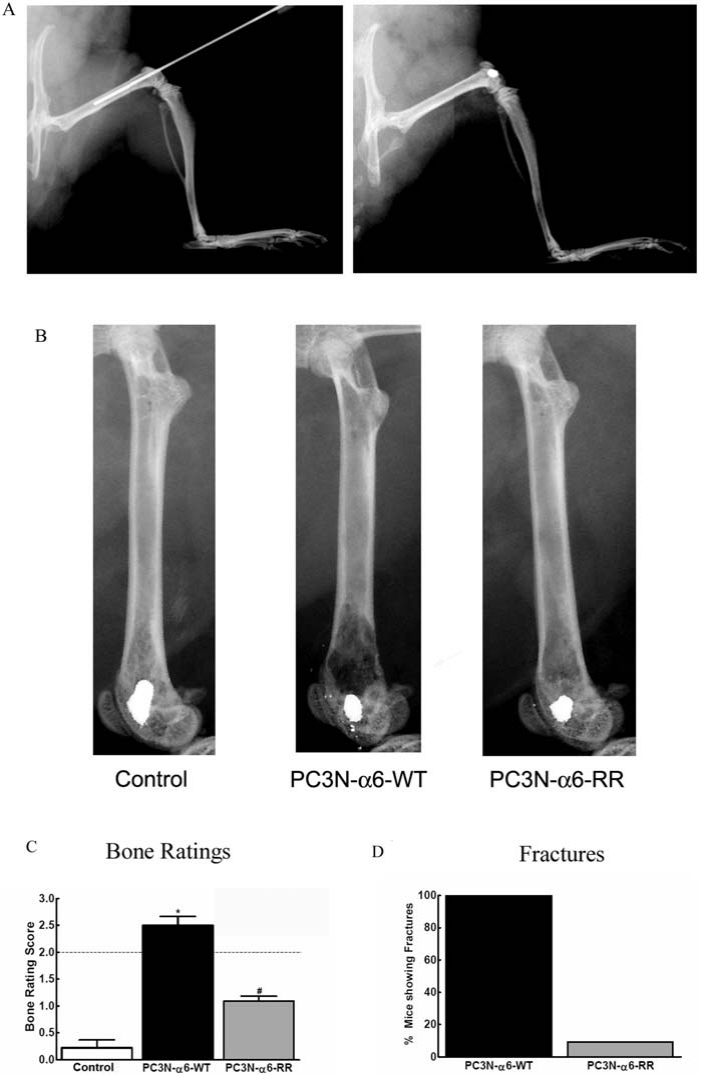
SCID Mouse xenograft model and quantification of bone destruction after injection of prostate cancer cells into the femoral intramedullary space. (A) Radiographic images were taken to verify needle placement inside the intramedullary space of the femur up to the distal third of the bone (left panel). The tumor cells were sealed into the bone (right panel). (B) Radiographic images taken at 21 days after injections indicating bone loss either from untreated mice (Control) or mice injected with tumor cells expressing the wildtype integrin (PC3N-A6-WT) or the mutated integrin (PC3N-A6-RR). (C) Quantification of bone loss in animals either sham injected (Control) or injected with tumor cells expressing the wildtype integrin (PC3N-A6-WT) or the mutated integrin (PC3N-A6-RR). Bone loss was rated according to the following 5 point scale: 0 = normal, 1 = bone loss observed in less than half of the distal third of the bone, 2 = bone loss observed in more than half of the distal third of the bone, no fracture, 3 = full thickness unicortical bone loss indicating unicortical bone fracture, 4 = full thickness bicortical bone loss indicating bicortical bone fracture. (D) The percent of mice with fracture 21 days following surgery. The number of mice in each group was twelve.

In order to determine the effect of human prostate cancer cells within the bone, we adapted the Clohisy-Mantyh murine model in which cancer cells are directly injected and sealed into the femur of a mouse[25]. Male SCID mice were anesthetized with ketamine/xylazine and an arthrotomy was performed exposing the condyles of the distal femur as previously described[26]. A hole was drilled into the femur for the injection needle to ensure accurate placement of tumor cells within the bone. The exact placement of the needle into the intramedullary space of the femur was confirmed by imaging (**Fig. 2a, left panel**). Human prostate tumor cells were injected into the right leg of the mouse and the injection site sealed with dental amalgam (**Fig. 2a, right panel**).

We next determined the deleterious effects of tumor cells residing within the bone using standard radiographic imaging in live animals 21 days following surgery. All animals injected with PC3N-A6-WT, and PC3N-A6-RR cells developed bone loss 21 days following surgery (**Fig. 2b**). Images consistently showed osteolytic activity, particularly of the metaphyseal bone at the distal (knee) end of the femur. Mice injected with the PC3N-A6-WT cells showing dramatically more bone loss compared to those injected with the PC3N-A6-RR cells. No bone loss was observed in animals injected with media alone (Fig 2b). Animals injected with the PC3N-A6-WT cells showed increased bone loss compared to those injected with PC3N-A6-RR cells (**Fig. 2b**). The radiographs were rated according to a 4 point scale in which 0 indicates normal bone and 3 indicates full thickness bicortical bone loss (**Fig. 2c**). Animals injected with PC3N-A6-WT cells showed a dramatic increase in fractures (unicortical or bicortical) 21 days following surgery compared to the PC3N-A6-RR treated mice (**Fig. 2d**). These data indicate that the placement of tumor cells within the bone containing a non-cleavable A6 integrin results in a significant delay in the development of bone loss.

**Figure 3:**
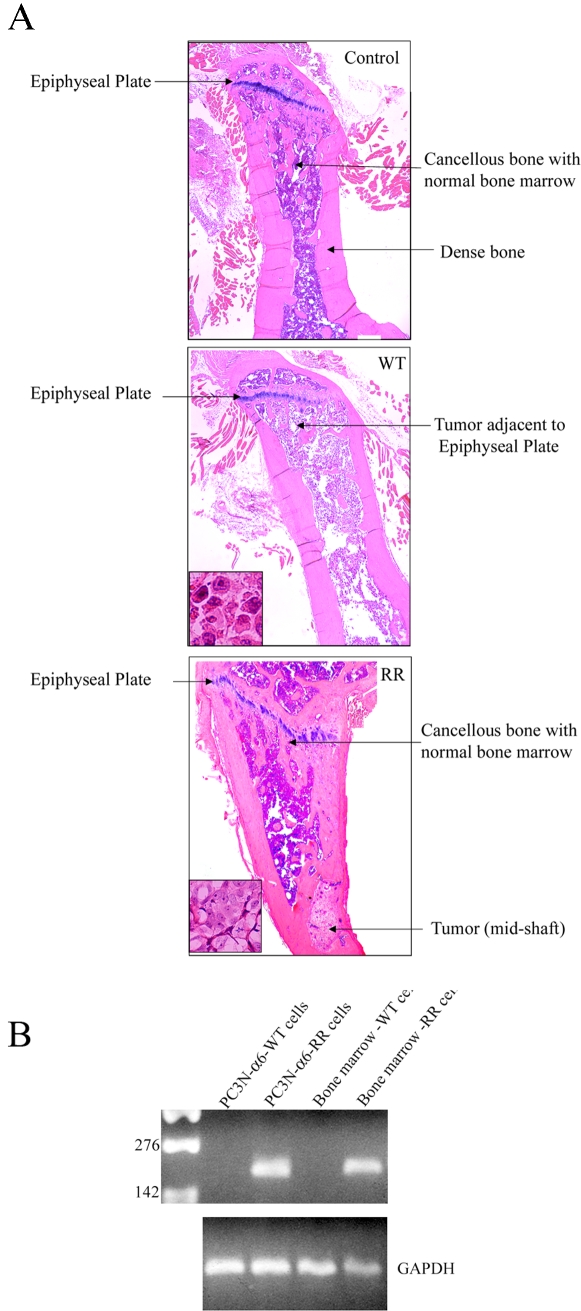
Histological examination of bone destruction, tumor cell distribution and verification of mutated integrin expression after injection of prostate tumor cells. (A) Hematoxylin-eosin staining of the normal bone (control) or bone injected with PC3N-A6-WT cells (WT, middle panel and inset) and PC3N-A6-RR cells (RR, bottom panel and inset). The growth plate of the bone (epiphyseal plate) is oriented at the top left of each panel for comparison purposes. (B) RT-PCR analysis to detect expression of the mutated 6 integrin in the bone marrow. Twenty one days following injection, bone marrow was harvested, RNA was extracted and analyzed. RNA from PC3N-A6-WT cells and PC3N-A6-RR cells growing in tissue culture was compared to the bone marrow isolated from mice injected with PC3N-A6-WT cells (Bone marrow-WT cells) or PC3NA6- RR cells (bone marrow-RR cells). GAPDH amplification was carried out as control and the kB markers are as shown.

Although the imaging results provided information about severe bone destruction, it did not give direct information about the distribution of the tumor cells. Bone loss is a consequence in part of tumor cells resident within the bone; an event that dramatically affects the critical balance of osteolytic and osteoblastic activity[3]. We directly investigated the distribution of the tumor cells within the bone using histological analysis by hemotoxylin and eosin staining of decalcified specimens. The mouse bones were carefully oriented for longitudinal sectioning to include observing the epiphyseal plate as well as the distal region of the bone in the same section (**Fig. 3A, top panel**). The analysis of the bones from the tumor injected animals demonstrated presence of tumor in the entire intramedullary space of the bone in those injected with PC3N-A6-WT cells along with invasion into the cortical bone (**Fig. 3A, center panel**). The tumor cells containing the cleavable integrin had reached the epiphyseal plate; the bone marrow was completely replaced. This result is consistent with the knowledge that once prostate cancer has established itself in bone marrow it will eventually replace the marrow, interrupting bone homeostasis[3]. In contrast, PC3N-A6-RR injected animals contained tumor cells in the mid-shaft region of the bone and the tumor failed to reach the epiphyseal plate. Normal bone marrow was present in the areas that did not contain tumor cells (**Fig. 3A, bottom panel**). The tumor cells within the mid shaft region or those that had reached the epiphyseal plate were viable and morphologically indistinguishable. (**Fig. 3A, center and bottom panel, insets**). The tumor distribution pattern was found to be consistent in the histological analysis of all the test animals assayed.

In order to confirm that the injected tumor cells were expressing the mutated integrin, 21 days following injection of PC3N-A6-WT or PC3N-A6-RR cells, the marrow was expressed from the intramedullary space of the mouse femurs. Analysis of bone marrow samples resulted in mRNA specific for the PC3N-A6-RR cells in marrow from mice injected with PC3N-A6-RR, but not PC3N-A6-WT mice. These data indicated that PC3N cells transfected with the uncleavable A6 integrin maintained expression of the mutant integrin within the intramedullary space of the femur and were present 21 days following injection of the cells into the femur (**Fig. 3B**).

**Figure 4:**
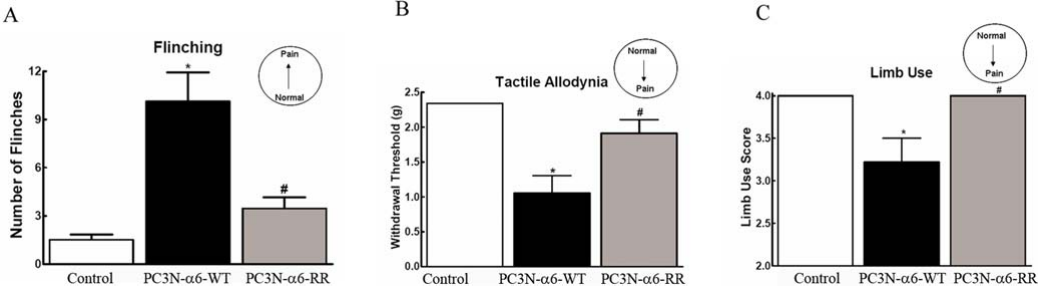
Development of cancer-induced spontaneous pain, tactile allodynia, and movement-evoked pain in animals 21 days after surgery. (A) Spontaneous pain as measured by flinching of the ipsilateral hindlimb was determined in sham injected animals (control) or those injected with tumor cells expressing the wild type integrin (PC3N-A6-WT) or those injected with tumor cells expressing the mutated integrin (PC3N-A6-RR). Elevation in flinching behavior is indicative of an increased pain response. (B) Tactile allodynia as measured by paw-withdrawal from von Frey filaments in sham injected animals (Control) or those injected with tumor cells expressing the wild type integrin (PC3N-A6-WT) or those injected with tumor cells expressing the mutated integrin (PC3N-A6-RR). A decrease in the withdrawal threshold is indicative of an increased pain response. (C) Movement evoked pain was observed in sham injected animals (control) or those injected with tumor cells expressing the wild type integrin (PC3N-A6-WT) or those injected with tumor cells expressing the mutated integrin (PC3N-A6-RR).

Behavioral analyses of spontaneous and evoked pain were determined 21 days following injection of PC3N-A6-WT or PC3N-A6-RR cells into the femur to evaluate the role of cleavage of A6 integrin on the development of spontaneous and evoked cancer pain behaviors. Spontaneous pain was measured by assessing flinching of the cancer treated hind limb as previously described[26]. Mice injected with PC3N-A6-WT cells showed increased spontaneous flinching behavior compared to PC3N-A6-RR treated mice which demonstrated low levels of flinching that were comparable to control animals (**Fig. 4a**). Evoked pain, as indicated by tactile allodynia, was determined by paw withdrawal from probing of the hind paw ipsilateral to the cancer treated femur with calibrated von Frey filaments as previously described[26]. Mice injected with the PC3NA6-WT cells showed tactile allodynia as indicated by lowered threshold for paw withdrawal from von Frey filaments (**Fig. 4b**). In contrast, PC3N-A6-RR injected mice did not show tactile allodynia, with paw-withdrawal thresholds similar to control animals (**Fig. 4b**). Movement-evoked pain was determined by rating the limb use during normal ambulatory movement as previously described[26]. Mice injected with PC3N-A6-WT cells developed movement evoked pain whereas mice injected with PC3N-A6-RR cells showed no movement-evoked pain, with limb use ratings the same as control animals (**Fig. 4c**).

These data indicate that cleavage of integrin A6 influenced the development of cancerinduced spontaneous and evoked pain associated with bone loss and fracture. Preventing cleavage of integrin A6 dramatically reduced bone loss and development of cancerinduced pain. In contrast, expression of the cleavable A6 integrin increased cancerinduced bone loss and fracture with a concurrent increase in pain behavior.

## Discussion

In this study we exploited a direct injection bone model to specifically place human prostate tumor cells directly into the physiologically and clinically relevant bone environment, rich in laminin 332 and 511. This allowed us to determine if blocking production of a laminin receptor (A6pB1) would alter the ability of the tumor cells to migrate or modify the environment within the bone and whether this held functional significance in terms of bone pain.

The results showed that modifying the production of the A6pB1 laminin receptor on the tumor cell surface can remarkably hinder migration within the bone. The direct injection model allows for the precise placement of known numbers of tumor cells within the bone. Since the tumor cells grow equally well independent of integrin status [13], [27] (both in tissue culture and as human tumors in mice), the data suggests that the distribution of tumor cells within bone may be a key element influencing cancer induced bone pain. The inability of tumor cells to reach the epiphyseal region of the bone due to integrin status coincided with a remarkable decrease of three distinct behavioral pain measurements. While it is unknown currently whether location of tumor cells within bone and bone pain were functionally linked, other reports have indicated that within bone, microenvironments are sufficiently distinct to warrant this possibility[28].

It is possible that the diminished bone pain is due to the inability of the integrin to be cleaved, independent of the location of the tumor cells within the bone. Integrins and cadherins are cleaved or shed on tumor cell surfaces[29]–[32]. The liberated fragments of receptors undergoing ectodomain cleavage are biologically active and may contribute to cancer induced bone pain[33]. Further experiments will distinguish between these possibilities.

Although bone metastases are traditionally thought to be either osteolytic or osteoblastic, morphological analyses have revealed that, in most patients, bone metastases have both osteolytic and osteoblastic elements[38]. In prostate cancer patients, bone metastases frequently show more osteoblastic phenotype, although some patients have osteolytic lesions similar to those seen in patients with metastatic breast cancer[38]. This research focused on a human prostate cancer cell line that produced mostly osteolytic bone remodeling in the mouse bone. The inability of the integrin to be cleaved resulted in reduced bone loss and fracture. As most bone loss and fracture are observed at the distal end of the bone, the inability of the tumor cells to reach the epiphyseal region of the bone may have reduced the bone loss and fracture in that area. Future studies on the role of integrins on osteoblastic bone remodeling would be needed to determine whether altering integrin function will also alter osteoblastic reactions in the bone.

Finally we note that cancer induced bone destruction is associated with severely reduced quality of life stemming from complications such as hypercalcemia, bone fracture and incapacitating pain. Advances in cancer detection and therapy are extending the life expectancy of cancer patients and is increasing a focus on improving patients' quality of life. Both the use of the novel xenograft mouse model for studying the prevention of cancer induced bone pain and targeting adhesion receptors on the tumor cell surface for the bone should significantly impact the quality of life for patients with metastatic disease.

## Materials and Methods

### Human Prostate Cancer Cell Lines

The PC3N cells were transfected with plasmids containing either the wildtype or mutated integrin A6 as previously described[13]. The stable clones (PC3N-A6-WT expressing wild-type integrin A6 and PC3N-A6-RR expressing the non-cleavable integrin A6) were isolated and maintained at 37°C in a humidified atmosphere of 95% air and 5% CO_2_. PC3N-A6-WT and PC3N-A6-RR cells were grown in Iscove's Modified Dulbecco's Medium (IMDM) (Gibco BRL, Gaithersburg, MD, USA) plus 10% fetal bovine serum (FBS) in presence of 6 g/ml Blasticidin (Invitrogen Corporation, Carlsbad, CA, USA) and 1 g/ml Zeocin (Invitrogen Corporation, Carlsbad, CA, USA).

### Antibodies and Chemicals

The antibodies were as follows: J1B5, rat monoclonal anti- A6 integrin (Dr. Caroline Damsky, University of California, San Francisco, USA)[34]; AA6A rabbit polyclonal anti-A6 integrin antibody[13] and AIIB2, rat monoclonal anti- B1 integrin antibody[35] (American Type Culture Collection). Human recombinant EGF was purchased from Invitrogen Corp., Carlsbad, CA, USA. Urokinase was purchased from Chemicon (Temecula, CA, USA).

### Immunoblotting

Cells were grown to confluency and then washed three times with HEPES buffer and lysed in cold RIPA buffer plus protease inhibitors (PMSF, 2 mM; leupeptin and aprotinin, 1 g/ml). The lysates were briefly sonicated on ice before being suspended in 2× non-reducing sample buffer. Samples were boiled for 5 min, and after a quick chill on ice, they were loaded onto 7.5% SDS-PAGE. Proteins resolved in the gel were electrotransferred to Millipore Immobilon-P polyvinylidene fluoride (PVDF) membrane (Millipore, Bedford, MA, USA), incubated with Western blotting antibodies plus secondary antibody conjugated to horseradish peroxidase and visualized by chemiluminescence (ECL Western Blotting Detection System, Amersham, Arlington Heights, IL, USA).

### Matrigel Migration Assay

Six well plates were coated with 1 ml Matrigel (BD Biosciences, Bedford, MA) and supplemented with laminin 332 containing conditioned medium from HaCaT cells in 2∶1 ratio and allowed to solidify. A sterile circular coverslip was placed in the center. Cells were then seeded in the plates and allowed to adhere for 2 hrs at 37 degrees C. Any non-adherent cells were removed and then coverslips were removed leaving a clear zone in the center of the plate. The periphery of this zone was marked. Serum free media or serum free media with blocking antibody was added to the cells and allowed to incubate at 37 degrees C for 18 hours. 1 ng/ml EGF was added to the media to induce migration. The cells were observed using a Zeiss Axiovert microscope (2.5× magnification) and images were collected using a CCD camera.

### Surgery

All procedures involving animals were approved by the University of Arizona institutional animal care and use committee, IACUC protocol #06-081. Animals were anesthetized with ketamine/xylacine and an arthrotomy was performed exposing the condyles of the distal femur. A hole was drilled into the femur, and the needle was inserted into the hole. Faxitron images were taken on 2 planes to verify needle placement inside the intramedullary space of the femur. PC3N-A6-WT, or PC3N-A6-RR cells, 10_6_ in 5 ul of filtered minimal essential medium (MEM) containing 1% bovine serum albumin (BSA), or 5 ul of filtered MEM containing 1% BSA alone (control) was injected into the intramedullary space of the mouse femur of the right leg and the injection site was sealed with dental amalgam. For the following experiments 12 animals received PC3N-A6-WT, 12 received PC3N-A6-RR cells, and 12 animals received cell-free serum (control).

### Behavioral Pain Measures [26]

*Movement-evoked pain*. The mouse was placed in an empty mouse pan and observed while walking across the pan in a continuous motion. Limping and/or guarding behavior of the right (cancer injected) hindlimb was rated on the following scale: 0 = complete lack of use, 1 = partial non-use, 2 = limping and guarding, 3 = limping, 4 = normal walking. *Spontaneous pain*. To assess flinching and guarding behavior, animals were placed in raised plexiglass chambers with a wire grid floor for observation of flinching and guarding of the right hindlimb. The mice were allowed to acclimate to the chamber for 20 minutes. Guarding and flinching behaviors of each mouse were measured for 2 minutes. The number of flinches was counted, and the time spent guarding the foot (the foot is lifted off of the floor) was measured. *Tactile hypersensitivity (allodynia)*. Paw withdrawal thresholds from von Frey filaments were determined in the manner described by Chaplan et al.[36] Paw withdrawal threshold was determined in response to probing with calibrated von Frey filaments. The mice were kept in suspended cages with wire mesh floors and the von Frey filament was applied perpendicularly to the plantar surface of the paw of the mouse until it buckled slightly, and was held for 3–6 sec. A positive response was indicated by a sharp withdrawal of the paw. The 50% paw withdrawal threshold was determined by the non-parametric method of Dixon[37]. An initial probe equivalent to 2 g was applied and if the response was negative, the stimulus was incrementally increased until a positive response was obtained, then decreased until a negative result was obtained. This up-down method was repeated until 3 changes in behavior were determined, and the pattern of positive and negative responses was tabulated. The 50% paw withdrawal threshold was determined as (10_[Xf+k*]_) /10,000, where Xf = the value of the last von Frey filament employed, k = Dixon value for the positive/negative pattern, and * = the mean (log) difference between stimuli. All pain tests were performed on each animal in the following order, movement-evoked pain, spontaneous pain (after the 20 min acclimation period), and tactile hypersensitivity.

### Determination of bone destruction

Radiographs were taken prior to the injection of the cancer cells (baseline, BL) and 21 days following induction of bone cancer using a Faxitron MX-20 machine at 7 uM nominal resolution with an X-ray current of 300 uA and a voltage of 26 kV (Faxiton X-ray Corp., Wheeling, IL). Digital radiographs were taken following behavioral testing on 12 animals per treatment condition. Images were captured by a digital camera and transferred to computer and saved in digital grayscale format (TIFF). Bone loss was rated according to the following 4 point scale: 0 = normal, 1 = bone loss, no fracture, 2 = full thickness unicortical bone loss indicating unicortical bone fracture, 3 = full thickness bicortical bone loss indicating bicortical bone fracture.

### Statistical analyses

Statistical comparisons between treatment groups were done using ANOVA. Pairwise comparisons between groups were made with Student's t-test, multiple comparisons between groups were done using Newman-Keuls Multiple Comparison Test. For the rating assays, limb use and bone loss, statistical comparisons were made using non-parametric analysis with the Mann-Whitney test. For all analyses, significance was set at p<0.05.

### Histological analysis of tumor within the bone

On day 21 post-surgery, mice were deeply anesthetized with ketamine and perfused intracardially with 25 ml of 0.1 M PBS followed by 25 ml of 10% neutral buffered formalin. Ipsilateral femora were removed from 6 animals per condition and postfixed overnight in 10% neutral buffered formalin. Femora were rinsed in water to remove formalin prior to being placed in Decal solution (RDO-Apex, Aurora, IL), for an hour to achieve decalcification. Samples were dehydrated in graded ethanol solution before carefully embedding in paraffin. They were oriented so that the entire length of the femur could be longitudinally sectioned. Sections 3 um thick were stained with hematoxylin and eosin to visualize normal marrow elements and cancer cells under bright field microscopy. The specimens were observed using a Nikon microscope (10× magnification) and images were collected using a CCD camera.

### RT-PCR analysis of PC3N-A6-RR cells in the intramedullary space of the femur

Bone marrow was extracted from the bones of 6 animals per treatment condition by flushing bone cavity with PBS. Total RNA was obtained from bone marrow and PC3N-A6-WT and PC3N-A6-RR cells by extraction with TRIzol reagent (Invitrogen Corp., Carlsbad, CA, U.S.A.) according to the manufacturer's protocol. RNA samples were evaluated for integrity of 18S and 28S rRNA by ethidium bromide staining of 1 mg of RNA resolved by electrophoresis on a 1.0% agarose/formaldehyde gel. CDNA was prepared using High Capacity cDNA Archive kit (Applied Biosystems, Foster City, CA, U.S.A.) as per manufacturer's instructions. Our primers targeted the mutation (R594R595 to A594A595) in the PC3N- 6-RR integrin 6 and were 5′-CTC TGC TGC GCG AGT GAA TTC-3′ and 5′-TGT CTT GAT TTC CTT CTC GGG T-3′ (150 bp). The GAPDH primers used were 5′-TGG TAT CGT GGA AGG ACT CAT GAC-3′ and 5′-AGT CCA GTG AGC TTC CCG TTC AGC-3′ (181 bp). PCR products were electrophoresed on a 2% agarose gel and visualized with Ethidium Bromide staining.
